# The role of the pulmonary veins on left atrial flow patterns and thrombus formation

**DOI:** 10.1038/s41598-024-56658-2

**Published:** 2024-03-11

**Authors:** Jordi Mill, Josquin Harrison, Marta Saiz-Vivo, Carlos Albors, Xabier Morales, Andy L. Olivares, Xavier Iriart, Hubert Cochet, Jerome Noailly, Maxime Sermesant, Oscar Camara

**Affiliations:** 1 Physense, BCN Medtech, Department of Information and Communication Technologies, Universitat Pompeu Fabra, 08018 Barcelona, Spain; 2 Inria, Université Côte d’Azur, Epione team, 06902 Sophia Antipolis, France; 3 IHU Liryc, CHU Bordeaux, Université Bordeaux, Inserm, 33600 Pessac, France; 4 Bordeaux University Hospital, 33600 Bordeaux, France

**Keywords:** Fluid dynamics, Cardiology, Arrhythmias, Atrial fibrillation

## Abstract

Atrial fibrillation (AF) is the most common human arrhythmia, forming thrombi mostly in the left atrial appendage (LAA). However, the relation between LAA morphology, blood patterns and clot formation is not yet fully understood. Furthermore, the impact of anatomical structures like the pulmonary veins (PVs) have not been thoroughly studied due to data acquisition difficulties. In-silico studies with flow simulations provide a detailed analysis of blood flow patterns under different boundary conditions, but a limited number of cases have been reported in the literature. To address these gaps, we investigated the influence of PVs on LA blood flow patterns and thrombus formation risk through computational fluid dynamics simulations conducted on a sizeable cohort of 130 patients, establishing the largest cohort of patient-specific LA fluid simulations reported to date. The investigation encompassed an in-depth analysis of several parameters, including pulmonary vein orientation (e.g., angles) and configuration (e.g., number), LAA and LA volumes as well as their ratio, flow, and mass-less particles. Our findings highlight the total number of particles within the LAA as a key parameter for distinguishing between the thrombus and non-thrombus groups. Moreover, the angles between the different PVs play an important role to determine the flow going inside the LAA and consequently the risk of thrombus formation. The alignment between the LAA and the main direction of the left superior pulmonary vein, or the position of the right pulmonary vein when it exhibits greater inclination, had an impact to distinguish the control group vs. the thrombus group. These insights shed light on the intricate relationship between PV configuration, LAA morphology, and thrombus formation, underscoring the importance of comprehensive blood flow pattern analyses.

## Introduction

Atrial fibrillation (AF) is considered the most common arrhythmia in humans and is currently seen as a marker of an increased risk of stroke since it favours thrombus formation inside the left atrium (LA). AF produces 25% of acute ischaemic strokes in patients older than 80 years of age^1^. Moreover, ischaemic strokes in AF patients have been associated with worse outcomes when compared with patients without this type of arrhythmia^1^. According to recent studies, more than 90% of thrombi in non-valvular AF are formed in the left atrial appendage (LAA), a muscular ear-shaped cavity in the LA^2,3^.

LAA shapes are complex and variable among the general population; researchers have sought to classify LAA morphologies and relate them to the risk of thrombus formation. The most adopted classification includes four LAA shape categories^4^: (1) Chicken-wing (CW); (2) Cactus; (3) Windsock; and (4) Cauliflower. The CW LAA morphology is thought to provide more protection against thrombus formation. However, a consensus among the community has not been reached on the usefulness of such classification since it is based on qualitative descriptions of the LAA shape. Furthermore, Bai et al.^5^ found statistically significant bias in LAA morphology classifications by operators when using different imaging modalities. In consequence, new morphologies such as the seahorse shape^6^ have been proposed, as well as new ways to classify LAAs, as the one proposed by Yaghi et al.^7^ that is based on the angulation of the LAA’s base with respect to the tip. Despite a substantial amount of clinical and physiological research, a plausible explanation of the relationship between the LAA morphologies and thrombus formation is still missing. To overcome this problem and improve thrombogenic risk and stroke sub-type stratification, the morphology of the LA/LAA has been characterised with multiple indices such as volumes and areas, width, and height of the LAA, ostium (e.g., interface between the main LA cavity and the LAA) parameters (minimum/maxim diameters), LAA bending angles and tortuosity, among others^8–10^.

One of the possible reasons for failing to link LA shape characteristics with thrombus formation is to study LA morphology independently of other factors, despite thrombus formation being a multi-factorial phenomenon. Following Virchow’s triad, low blood flow velocities and stagnation have been associated with the triggering of the inflammatory process and, therefore, the risk of thrombus generation^11^. A detailed analysis of in-vivo LA hemodynamics in 4D (i.e., 3D plus time) in AF patients is difficult due to the spatio-temporal resolution of current imaging modalities used in clinical practice (e.g., echocardiography). Advanced imaging techniques such as 4D flow magnetic resonance imaging (MRI), allowing a more complete blood flow analysis, are emerging, but they still provide limited information in the left atria^12,13^. In consequence, several computational modelling studies have focused on simulating LA blood flow patterns, but rarely investigating their relation with LA morphology^13–39^. Surprisingly, very few studies have considered the pulmonary veins (PV) as a factor for thrombus formation, despite PVs directly affecting LA hemodynamics^10,13,15,18,30,32,40–42^.

On the other hand, PV configuration and orientation have been proved to be a key player in radiofrequency ablation therapy (e.g., PV isolation) since they are a preferential origin of ectopic foci in atrial fibrillation^43^. Some large-scale studies have classified PV configurations into different anatomical categories^44^, but they have never been considered as a factor in thrombus formation. Moreover, there is high anatomical variability among the population, where most humans have 4 PVs but there are reported cases with 3, 5, 6 or even 7 PVs. Even more, the orientation of how the PVs are inserted into the LA can differ substantially from patient to patient.

The aim of the present work was to study the influence of the PV configuration in LA haemodynamics and its relation with thrombus formation, hypothesising that it will determine how the blood flow enters the LAA, allowing a better or worst blood washout. A large cohort of 130 AF cases, including follow-up information on which subjects developed LAA-based thrombus or suffered cerebrovascular accident/transient ischaemic attack (CVA/TIA) events, was analysed. At the time being, this represents the largest in-silico study with patient-specific LA fluid simulations performed so far, since most investigations available in the literature have only been applied to a limited number of patient-specific cases (see^45^ for a recent review), with only a few processing tens of cases^32,41,46^.

For this purpose, Computational Fluid Dynamics (CFD) simulations with Arbitrary Lagrangian-Eulerian (ALE) formulation were employed to investigate 130 patients with non-valvular atrial fibrillation, utilising laminar flow conditions and modelling a non-Newtonian fluid. The primary focus will involve extracting various anatomical features of the LA and the LAA, along with determining the inclination of the pulmonary veins. These morphological and blood flow pattern parameters served as critical indicators to discern potential disparities between patients exhibiting thrombus formation and those without. The detailed examination of these parameters aims to have valuable insights into the underlying factors associated with thrombus occurrence in non-valvular atrial fibrillation, paving the way for enhanced diagnostic precision and targeted therapeutic interventions.

## Results

### Morphological analysis

Table 1 lists the statistically different characteristics (i.e., morphological, angular, haemodynamic) between thrombus and non-thrombus groups after performing the statistical analysis. Supplementary Fig. A1 shows the distribution of our cohort concerning LA and LAA volumes, with the majority of cases having LAA volumes between 10 and 14 mL, and LA volumes between approximately 135 and 180 mL.

**Table 1:** Morphological and haemodynamic features of the left atria (LA) and pulmonary veins (PV), with statistical significance between control (C) and thrombus/stroke groups (T/S).

Feature	Control	T/S group	*p* value
$\gamma$ (degrees)	$37.29 \pm 37.24$	$35.35 \pm 31.56$	**<0.01**
Mean inclination (degrees): $1/4(\phi _{1} + \phi _{2}+ \phi _{3} + \phi _{4})$	$7.48 \pm 1.94$	$8.67 \pm 2.18$	**<0.01**
Inclination of the left side (degrees): $1/2(\phi _{3} + \phi _{4})$	$14.94 \pm 4.9$	$17.33 \pm 5.65$	0.019
Inclination of the right side (degrees): $1/2(\phi _{1} + \phi _{2})$	$3.98 \pm 2.02$	$4.66 \pm 1.81$	**<0.01**
D1 (mm)	$27.80\pm 5.80$	$29.90\pm 7.80$	**<0.01**
D2 (mm)	$19.4 \pm 4.6$	$21.40\pm 5.20$	**<0.01**
h_LAA_	$17.0 \pm 5.60$	$19.20\pm 5.80$	0.019
h_θ_	$36.10 \pm 7.10$	$35.40\pm 6.80$	0.531
LAA tortuosity	$0.39 \pm 0.08$	$0.42 \pm 0.06$	**<0.01**
LA volume (mL)	$172.20 \pm 80.10$	$153.0 \pm 68.20$	0.02
Ostium perimeter (mm)	$74.80 \pm 16.30$	$82.30\pm 22.20$	**<0.01**
Ostium area (mm^2^)	$411.80 \pm 185.40$	$500.20\pm 275.80$	**<0.01**
Ostium mean diameter (mm)	$23.68 \pm 4.99$	$25.80 \pm 6.90$	**<0.01**
LAA/LA ratio (%)	$7.90 \pm 3.19$	$6.76 \pm 2.63$	**<0.01**
LAA volume (mL)	$10.70 \pm 6.90$	$13\pm 7.70$	0.04
Bending	$115.1 \pm 21.70$	$117.30 \pm 17.60$	0.53
Ostium irregularity	$0.024 \pm 0.026$	$0.022 \pm 0.025$	0.47
LA length	$179.10 \pm 58.40$	$180.50 \pm 43.30$	0.42
LAA length	$42.10 \pm 11.70$	$42.10 \pm 12.30$	0.76
Curvature	$0.068 \pm 0.017$	$0.062 \pm 0.019$	0.02
GI	$0.94 \pm 0.05$	$0.96 \pm 0.03$	0.02
ECAP	$16.99 \pm 25.35$	$16.57 \pm 23.26$	0.20
OSI	$0.21 \pm 0.04$	$0.20 \pm 0.03$	0.61
TAWSS	$0.45 \pm 0.30$	$0.49 \pm 0.38$	0.53
Particle age	$1.29 \pm 0.23$	$1.32 \pm 0.23$	0.71
Total number of particles	$142 \pm 101.0$	$209.4 \pm 96.8$	**<0.01**
LAA shape			0.04
NA (n = 48)	40.3	33.4	–
A (n = 55)	33.8	53.7	–
CW (n = 27)	26	13	–
3 PV (n = 4)	3	1	–
4 PV (n = 66)	39	25	–
5 PV (n = 43)	23	19	–
6 PV (n = 13)	7	6	–
7 PV (n = 4)	3	1	–

Feature values are presented as mean ± SD or median (inter-quartile range). $\gamma$: angle between the LAA and the left superior PV main directions. $\phi _i$: inclination of the *i* PV. LIPV: left inferior pulmonary vein. LAA: left atrial appendage. D1, D2: maximum and minimum diameters of the ostium, respectively. GI: gyrification index. ECAP: endothelial cell activation potential. OSI: oscillatory shear index. TAWSS: time-averaged wall shear stress. A/NA: aligned/non-aligned LSPV and LAA. CW: chicken-wing LAA morphology. In bold the parameter with the most significance.

#### Left atrial geometrical parameters

The most significant morphological parameters to discern thrombus vs non-thrombus groups are shown in Table 1, such as the LAA ostium characteristics, which have already been reported in the literature as being associated to thrombogenic risk (i.e., larger ostia)^10^. Additionally, the LAA/LA ratio and the LAA tortuosity were significantly larger in thrombus cases. On the other hand, LA and LAA volumes, which have been reported to the literature as being good predictors of thrombus risk, were not as significant as the ones listed above.

#### Number and distribution of pulmonary veins

The distribution of the studied population with respect to the number of pulmonary veins and the incidence of cardiovascular incident or thrombus history (T/S) is shown in Table 1). The topological characterisation of the PV was further analysed regarding the number of PV in each side of the LA (left vs. right), resulting in the identification of eleven PV configurations, as depicted in Fig. 1. For instance, cases with 5 PV included LA geometries with 3 vs. 2 PV in the right and left side, respectively (left, third row in the figure), or 4 vs. 1 PV, which could in itself had two central PV with varying distance between them (middle and right examples in the third row of the figure). Most of the identified PV configurations are already described in the literature^47,48^, except the one with 3 PV on the left side in the 5 and 7 total PV group, which was present in two cases.

Patients with 5 and 6 PV presented the largest percentage of T/S cases (around 46% of cases), with a slightly smaller percentage in cases with 4 PV (37% of cases). Due to the reduced number of patients with 3 and 7 PV, the resulting percentages (25% of T/S cases) may not be representative. On the other hand, differences were found in the mean LA volume for each PV group. The mean LA volume was slightly higher in subjects with 6 and 7 PV (185 and 190 ml, respectively) than with 3, 4 and 5 PV (152, 161 and 166 mL, respectively).

#### Alignment of the pulmonary veins and left atrial appendage

The angles characterising LA topology were significantly different between control and thrombus/stroke cases, mainly the $\phi$ angles (e.g., PV inclination) from the left and right sides, which were larger in thrombus cases, i.e., having more inclined left and right PVs. In our cohort, the angle between the left PVs ($\beta$) varied less than the one between the right PVs ($\alpha$).

On the other hand, the $\gamma$ angles (e.g., alignment between the LAA and the left superior pulmonary vein - LSPV) were smaller in the T/S cases than in controls, i.e., the LAA was more aligned with the LSPV in the thrombus/stroke group. The LSPV-LAA alignment classification identified additional differences between the different groups. For instance, the aligned group (Group A) had more cases on the thrombus group. In contrast, the CW group had more cases on the control group, suggesting a protective role of this LAA morphology regarding thrombus formation.

### Left atrial blood flow patterns

**Figure 1:**
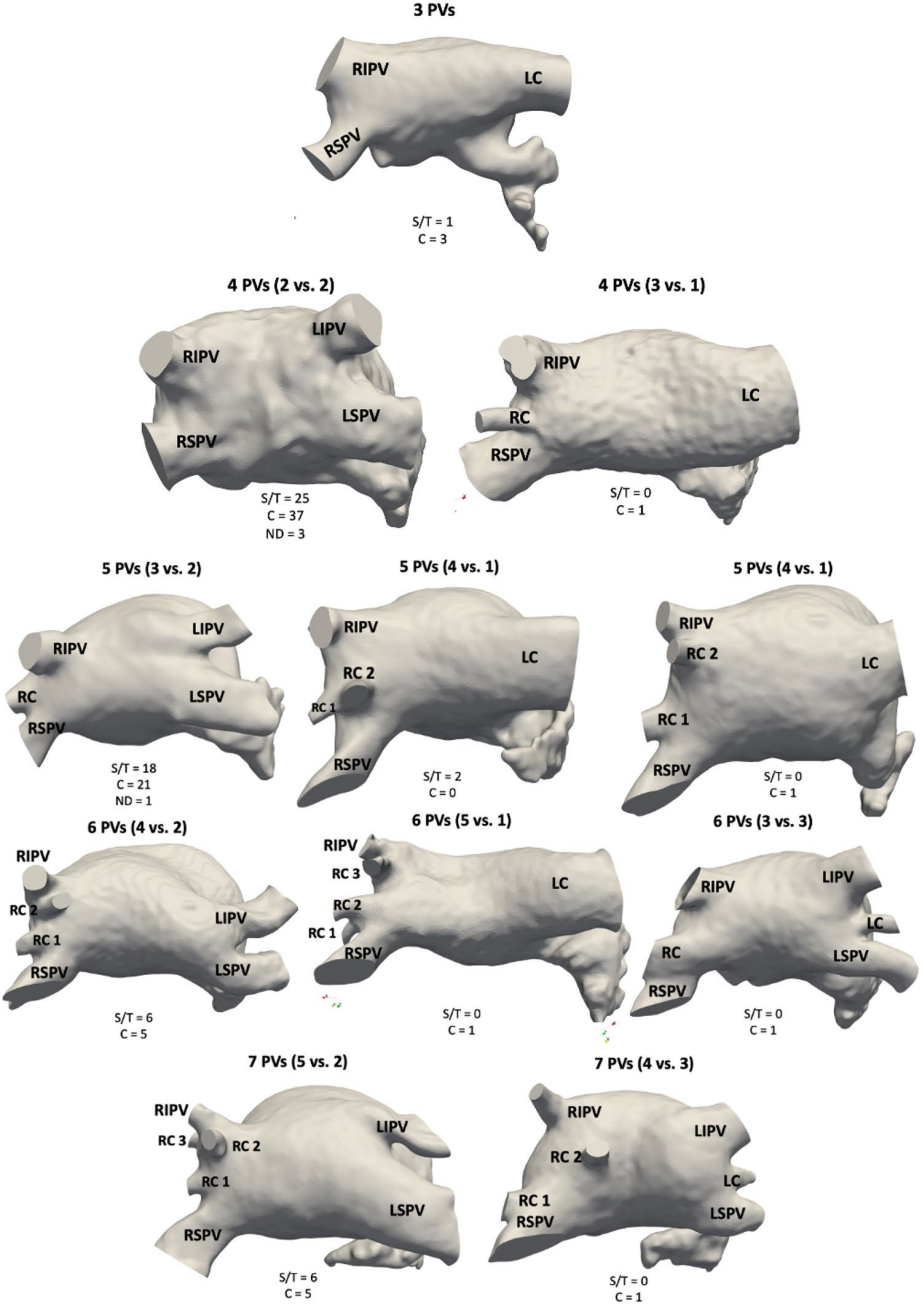
Left atrial configurations based on the topology of the pulmonary veins (PV). The eleven PV topological configurations found in the analysed cohort, depending on the total number of PV and its distribution on the right and left side of the left atria (in brackets for each configuration). The number of control (C) and thrombus/stroke (T/S) cases is also indicated in each topological configuration. RIPV/RSPV: right inferior/superior PV. LC/RC: left/right central pulmonary vein. LIPV/LSPV: left inferior/superior PV. RC1: right central PV that is closest to the RSPV; RC2: right central PV that is closest to the RIPV if there is no RC3; with an existing RC3, RC2 is in between RC1 and RC3; RC3: right central PV that is closest to the RIPV.

#### Blood flow patterns

**Figure 2:**
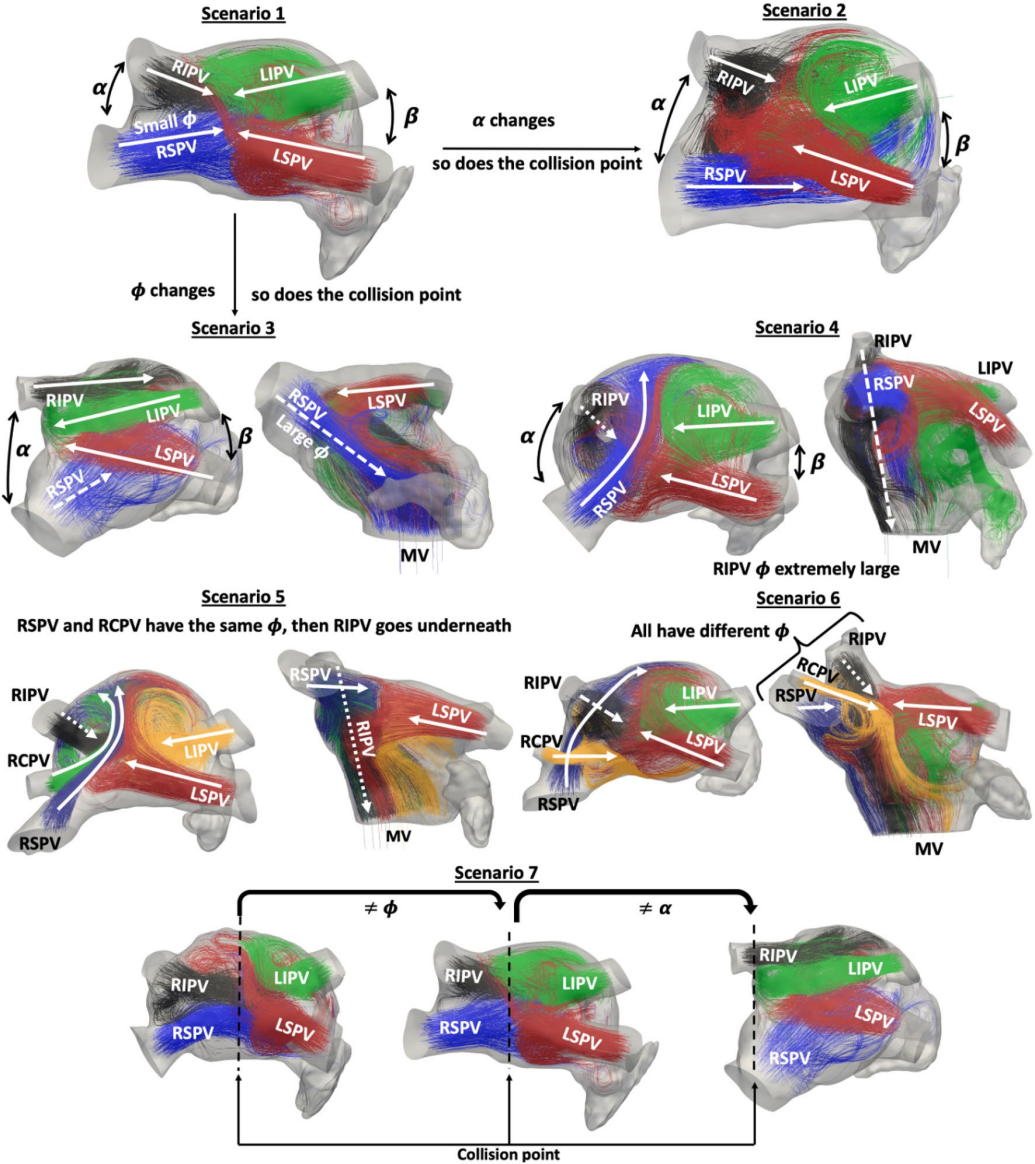
The role of the pulmonary vein configuration in left atrial blood flow patterns. Blood flow patterns in the left atria (LA) obtained in morphologies with different pulmonary vein (PV) configurations. PV configuration is characterised by several angles between the different sub-structures in the LA. Colours in streamlines indicate the PV origin of blood flow. Scenarios 1–4,7 (4 PV): green/red represents the left inferior/superior PV (LIPV/LSPV); black/blue represents the right inferior/superior PV (RIPV/RSPV). Scenario 5 (5 PV): orange/red represents LIPV/LSPV; black/blue/green represents right inferior/central/superior PV (RIPV/RSPV/RCPV). Scenario 6 (5 PV): green/red/black/blue/orange represents LIPV/LSPV/RIPV/RSPV/RCPV. The black dashed line depicts flow collision points. The solid white line is the direction of the flow coming from the PV, the white dashed line having a higher inclination (higher $\phi$). The snapshots were taken at end diastole when the maximum velocity of the A wave is reached, just before mitral valve (MV) closing. The cardiac phase showed in the figure is ventricular systole.

The simulated blood flow patterns indicated that the orientation of the pulmonary veins had an impact on LA blood flow patterns in the analysed cases, especially on the location of flow collisions in the LA. Figure 2 depicts the most predominant blood flow patterns with different PV configurations, indicating the PV origin with coloured streamlines, and showing angular heterogeneity between several cases.

The following qualitative observations were made after a careful visual analysis of the 4D blood flow patterns in all simulated cases:

- Individuals can have the same number of PV but having a different topology (e.g., PV positions and orientations) that substantially change blood flow patterns.
- The most common PV configuration was composed of 4 PV (2 right side vs. 2 left side), with the collision between left-right PV flows taking place at the centre of the LA. With a larger angle between the right PV ($\alpha$ angle), the right side flow could go laterally through the sides of the LA to reach the LAA (scenarios 1,2 and 3 in Fig. 2).
- The inclination of the right PVs with respect to the main LA cavity ($\phi$ angles) determined whether the blood went vertically down towards the MV or to the centre of the LA. Scenarios 3 and 4 in Fig. 2.
- In cases with more than 4 PV, the presence of the additional right central pulmonary vein (RCPV) shifted the right superior pulmonary vein (RSPV) towards the right LA wall. Consequently, the RSPV is in a less centred position. With the RSPV being shifted to a more transverse position with respect to the main LA body, the flow crossed the LA, preventing the remaining right PV flows to collide with the left side flow. Scenario five in Fig. 2.
- If the RCPV was located at a higher position than the RSPV, RCPV flow was the one colliding with the left PV flow, forcing RSPV flow to go under RCPV one. Scenario 6 in Fig. 2.
- If the right PVs were very inclined (e.g., high $\phi$ angle), the collision point was shifted towards the right side; on the other hand if the angle between the right PVs ($\alpha$ angle) was also large, then the collision was clearly shifted to the left side; flow from the left PV could then reach the right LA wall. Scenario 7 in Fig. 2.

Another important finding was that when blood flow from the left PVs collided at the superior part of the LA, due to their orientation, the blood flow was subsequently directed to the LAA ostium, which is usually located just under the LSPV. In consequence, the $\alpha$ and $\beta$ angles, which characterise the angular difference between the PV in the same LA side (left and right, respectively), had a considerable effect on the resulting blood flow patterns.

#### In-silico haemodynamic indices

The total number of particles in the LAA at the end of the simulation was the only in-silico haemodynamic index able to classify thrombus vs. non thrombus cases, as shown in Table 1, with significantly higher values for the former. Particle age, blood flow stagnation from the simulated flow rate or endothelial cell activation potential (ECAP) distributions did not present significant differences between the two studied groups.

Despite the different number of particles for controls and thrombus cases, patients with similar morphological characteristics were found in the two groups. To better identify the relationship between the estimated in-silico indices and LA topology, we independently analysed patients depending on their number of PVs and the alignment between the left superior pulmonary vein and the LAA (see Fig. 9).

Table 2 summarises the distribution of the studied LA shapes based on the number of PV and the LSPV-LAA alignment-based categories, including the number of particles in the LAA at the end of the fluid simulations and the percentage of thrombus cases. In general, we can observe an association between the number of particles in the LAA and the alignment between the LAA and LSPV main directions (except for cases with 5 PV), as quantified by the $\gamma$ angle: lower values of $\gamma$, thus more aligned LAA and LSPV (Group A), were linked to more flow particles entering the LAA, compared to low LSPV-LAA alignment cases (Group NA). However, such observation was not valid for chicken-wing LA morphologies due to their extreme variations in ostium positions, bending angles and tip directions (see Fig. 9), which lead to different LAA blood flow patterns than in non-CW morphologies.

The number of pulmonary veins fully determined the origin of the flow particles entering the LAA. Overall, in LA geometries with only 3 PV (i.e., only one PV in the left side), 76% of LAA particles came from the left side. Interestingly, the same situation occurred in cases with 7 PV, where the left side only had two PV (bottom left in Fig. 1, the left side representing 61% of LAA particles. The remaining LA morphologies (4–6 PV) had a more balanced left-right PV contribution into the particles particles entering the LAA (56.92%, 55.94%, 52.43% from the left side for 4, 5 and 6 PV, respectively). However, we did not find any relation between the number of PVs, the LAA particles and the risk of thrombus.

A more detailed analysis of the relation between LA morphological parameters, blood flow patterns and in-silico haemodynamic indices is given below for cases with 4, 5, and 6 pulmonary veins. Due to the small number of individuals with 3 and 7 PV (four in each group) in the analysed cohort, it was difficult to draw any meaningful conclusion on these groups (analysis available in Supplementary Tables A1 and A5.

**Table 2:** Result of the particle study, grouped by number of PVs and type of

**Results of the particle study**								
	**A**		**NA**		**CW**		**All groups**	
	% of T	Av. Part(T+C).	% of T	Av. Part(T+C).	% of T	Av. Part(T+C).	% of T	Av. Part(T+C).
3 PV	$100\%$	353.00	$0\%$	113.33	–	–	$25\%$	173.25
4 PV	$46\%$	230.16	$35\%$	128.33	$23\%$	147.50	$37\%$	170.66
5 PV	$57\%$	215.05	$45\%$	253.62	$33.33\%$	129.07	$47\%$	194.19
6 PV	$60\%$	215.60	$40\%$	176.20	$33.33\%$	192.00	$46\%$	195.00
7 PV	$0\%$	204.00	–	–	$33.33\%$	132.00	$25\%$	150.00

Distribution of cases based on the number of pulmonary veins (PV) and alignment of left atrial appendage (LAA) and left superior PV (LSPV) main directions. Av. P.: Average of particles in the LAA at the end of the simulation, including control and thrombus cases. C, control group; T/S, thrombus or stroke; A/NA, LAA and LSPV main directions are aligned/not-aligned; CW, chicken-wing; % of T, % of thrombus cases in that group. The four cases in our database without follow-up were not included in this study.

#### 4 pulmonary veins

In LA geometries with 4 PV, which constituted the most populated group, there were a larger percentage of thrombus cases in the aligned LSPV-LAA group than in the non-aligned one (46% and 35% for Group A and Group NA, respectively), as it can be seen in Table 2. The same trend can be observed with the average number of LAA particles, with a higher value in aligned vs non-aligned LSPV-LAA cases (230.16 vs. 128.33 particles, respectively). When we delve more deeply into the study of the group of individuals with four pulmonary veins, we can observe how the number of LAA particles in the aligned group was lower in controls than in thrombus cases (198.75 and 278.06 particles, respectively), further demonstrating the association between a higher thrombus risk and a higher number of LAA particles (Fig. 6). Interestingly, despite its supposedly protective role, 23% of the cases with a chicken-wing (CW) morphology were in the thrombus group (Table 2).

**Figure 3:**
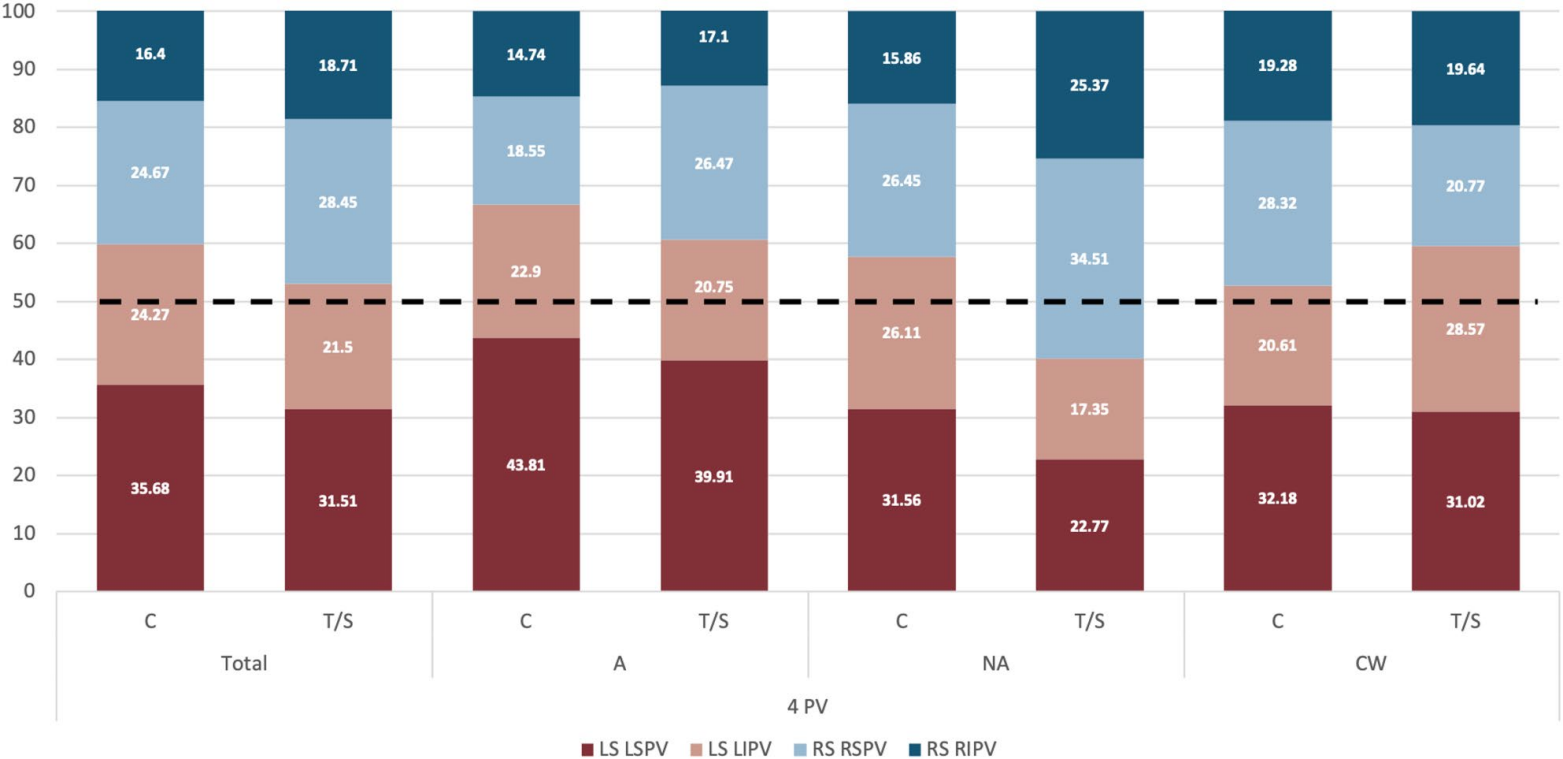
Pulmonary vein origin of simulated particles for cases with 4 pulmonary veins. Distribution of pulmonary vein origin of simulated particles reaching the left atrial appendage (LAA) in cases with 4 pulmonary veins (PV), including the control (C) and thrombus/stroke (T/S) groups and different types of LAA and left superior PV alignment. L/R: left/right; S/I: superior/inferior; A/NA: aligned/non-aligned; CW: chicken-wing. The warm and cold colours represents the PV from the left and right side, respectively.

Upon comparing the origin of blood flow entering the LAA between control and thrombus cases, no significant differences were observed in 4 PV cases when they were collectively assessed (Fig. 3 and first column Supplementary Table A2). However, when considering LSPV-LAA alignment sub-groups, distinctions emerged. In the aligned cases, the contribution of the left side PVs into flow entering the LAA was more dominant in controls comparing to thrombus cases (66.71% vs. 56.66% respectively). As shown in Fig. 3, this disparity was primarily driven by the reduction of LSPV contribution (43.81% vs. 39.91% in controls and thrombus cases, respectively), consequently increasing the role of the RSPV flow (18.55% vs. 26.47% in controls and thrombus cases, respectively). A similar pattern was observed in the non-aligned LA cases (Group NA), where an increased contribution from the left side was associated with a lower thrombosis risk (LSPV contribution of 57.67% and 40.12% in controls and thrombus cases, respectively). Moreover, in this scenario, the influence of the right side surpassed the left one in contrast to the aligned cases with 59.88% of the particles provided by the right side (refer to Fig. 3 and Supplementary Table A2).

Intriguingly, left atria with a chicken-wing LAA demonstrated an opposite trend compared to other LAA shapes, with control cases showing a greater contribution from the right PV (specifically, the RSPV) to LAA flow particles, as illustrated in Fig. 3. However, controls had fewer LAA particles than thrombus cases (147.5 and 190, respectively) following the previous trends.

In summary, the analysis of 4 PV cases suggests that:

- Higher LSPV-LAA alignment is related to having more particles in the LAA but not directly with a higher thrombogenic risk if not divided in sub-groups.
- Thrombus cases had more LAA particles within each LSPV-LAA alignment sub-group (e.g., in the aligned/non-aligned groups, controls have less LAA particles than thrombus cases).
- Having more LAA particles is related to a lower participation of the left PVs, except for CW group.
- Thrombus cases had a higher participation of the right PVs on LAA particles, in particular from the RSPV, except for CW group.
- Chicken-wing LAA morphologies present opposite trends than other LAA morphologies, thus need to be separately studied.

#### 5 pulmonary veins

In LA geometries with 5 PV, there were a larger percentage of thrombus cases in the aligned LSPV-LAA group than in the non-aligned one (57% and 45% for Group A and Group NA, respectively, both higher than in 4 PV cohort), as it was the case in the 4 PV group (see Table 2). The number of LAA particles effectively separated the controls and thrombus cases and the control cases in NA and CW LSPV-LAA alignment sub-groups. In the LSPV-LAA alignment group, the particles exhibit a notable ability to discriminate effectively between thrombus and the control group, displaying values of 190.55 and 169.45, respectively. Nevertheless, the inclusion of a single outlier distorts the mean particle count, resulting in a higher count for the control group in comparison to the thrombus group, yielding values of 190.55 and 207.57, respectively (see Supplementary Table A3 and Supplementary Fig. A2).

**Figure 4:**
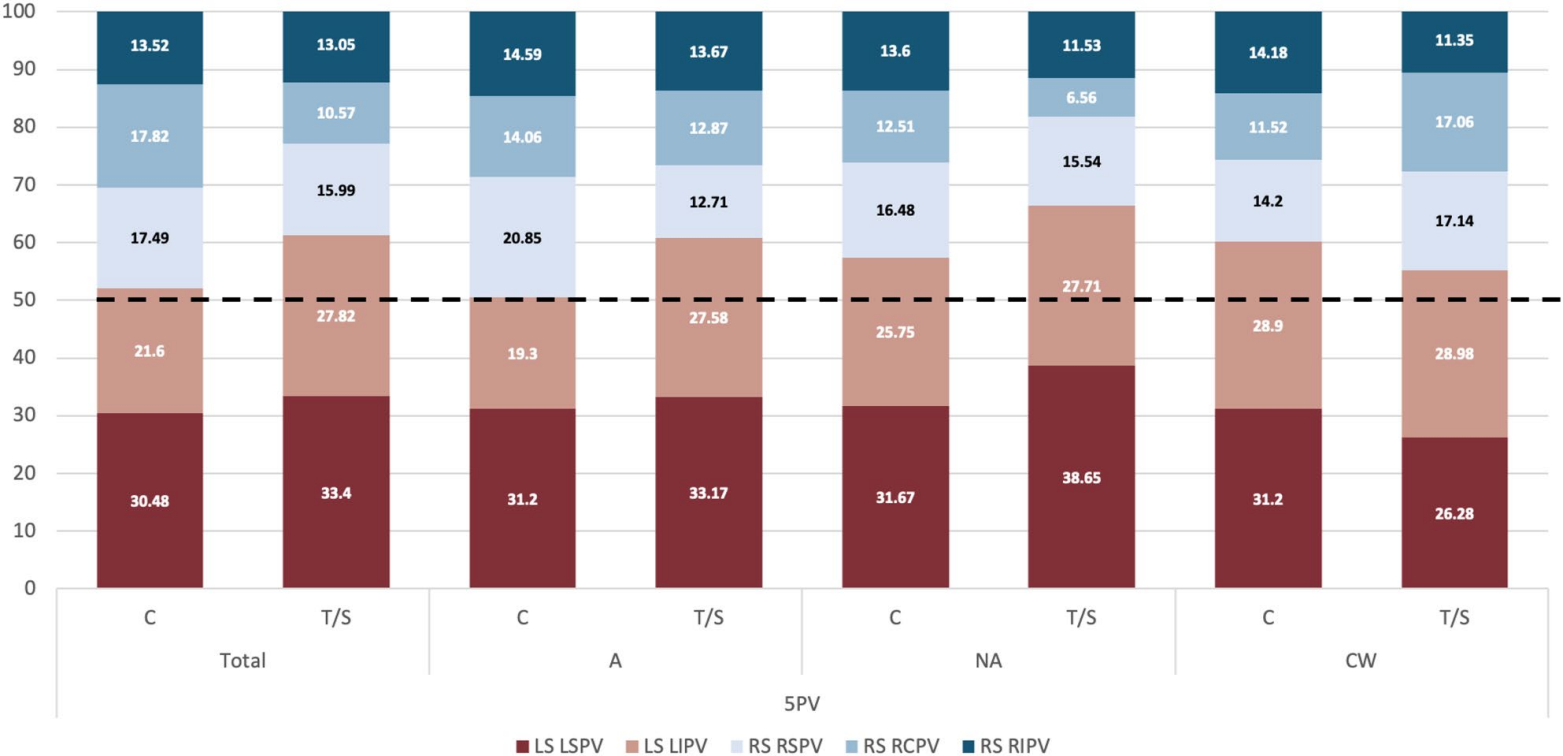
Pulmonary vein origin of simulated particles for cases with 5 pulmonary veins. Distribution of pulmonary vein origin of simulated particles reaching the left atrial appendage (LAA) in cases with 5 pulmonary veins (PV), including the control (C) and thrombus/stroke (T/S) groups and different types of LAA and left superior PV alignment. L/R: left/right; S/C/I: superior/central/inferior; A/NA: aligned/non-aligned; CW: chicken-wing. The warm and cold colours represents the PV from the left and right side, respectively.

A remarkable characteristic of the 5 PV group is that controls had a higher flow contribution of the right PV side in LAA particles than in thrombus cases, as illustrated in Fig. 6, which is the opposite behaviour found in 4 PV cases (i.e., higher left PV side contribution in controls). One potential reason for the different blood flow dynamics is the presence of the RCPV (the fifth PV), which spatially shifts the RSPV. Moreover, aligned LSPV-LAA cases had a lower number of LAA particles than the non-aligned ones (215.05 vs. 253.62 for Group A and Group NA, respectively), being again the contrary trend than in 4 PV cases (Table 2). Complementary, chicken-wing (CW) LAA cases with 5 PV and thrombus history had more LAA particles than controls (206.75 vs. 73.57, respectively). However, the contribution of the left and right PV sides into the flow entering into the LAA was similar for both groups in CW shapes. On the contrary, in A/NA groups, flow from the right PV is contributing more to LAA particles than the left one in controls (Fig. 4). In Group A, cases had a higher weight of the RSPV flow in LAA particles of controls than in thrombus cases (20.85% and 12.71%, respectively). In Group NA, the difference was only found in the RCPV (12.51% on average and 6.56 %, respectively).

#### 6 pulmonary veins

As in cases with a lower number of PV, LA geometries with 6 PV had a higher percentage of thrombus cases in LSPV-LAA aligned subjects vs non-aligned ones (60% vs. 40%, respectively). For all the sub-groups, the number of particles at the end of the simulations was a good indicator to distinguish control and thrombus group.

**Figure 5:**
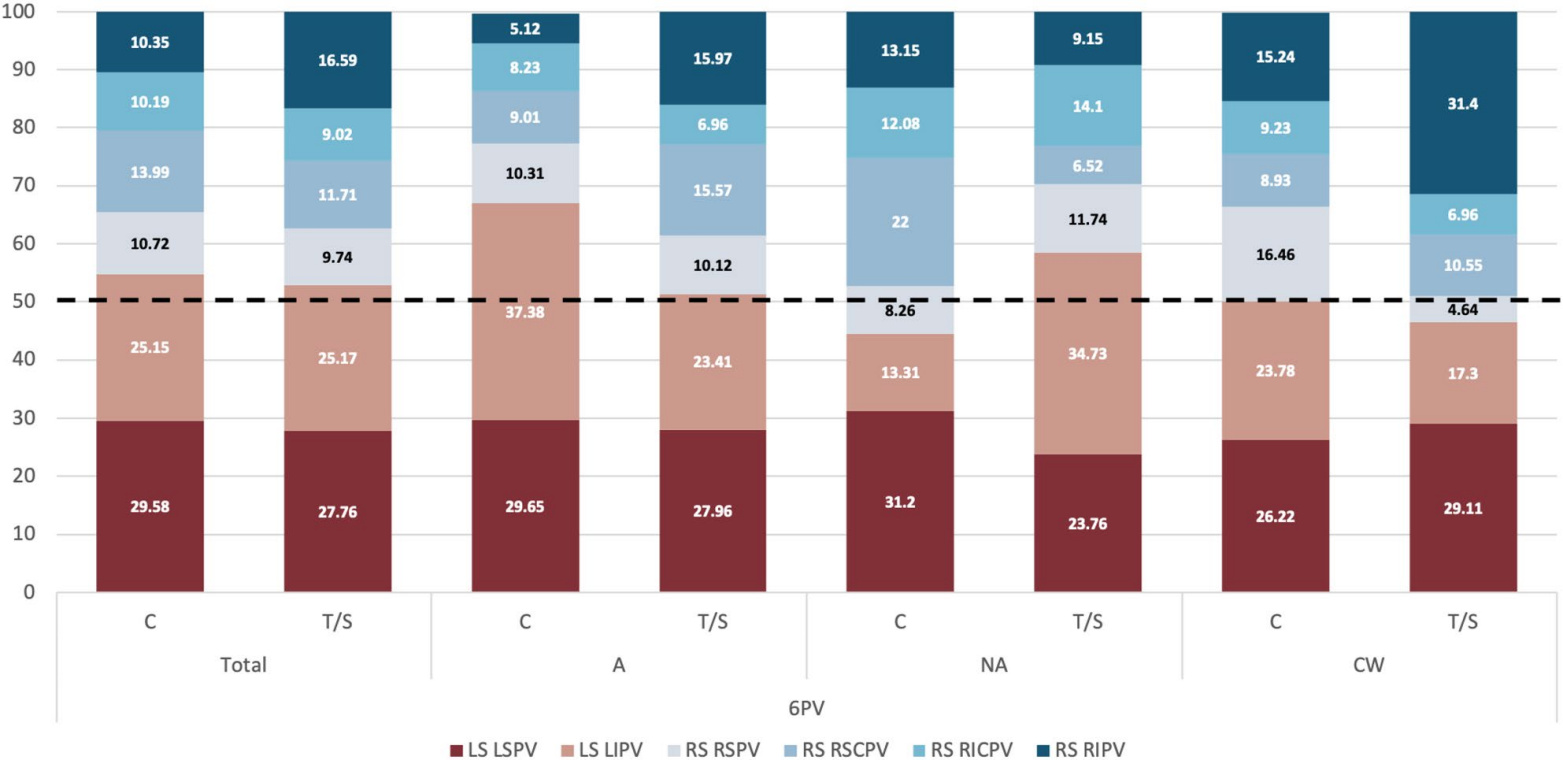
Pulmonary vein origin of simulated particles for cases with 6 pulmonary veins. Distribution of pulmonary vein origin of simulated particles reaching the left atrial appendage (LAA) in cases with 6 pulmonary veins (PV), including the control (C) and thrombus/stroke (T/S) groups and different types of LAA and left superior PV alignment. L/R: left/right; S/C/I: superior/central/inferior; A/NA: aligned/non-aligned; CW: chicken-wing. The warm and cold colours represents the PV from the left and right side, respectively.

**Figure 6:**
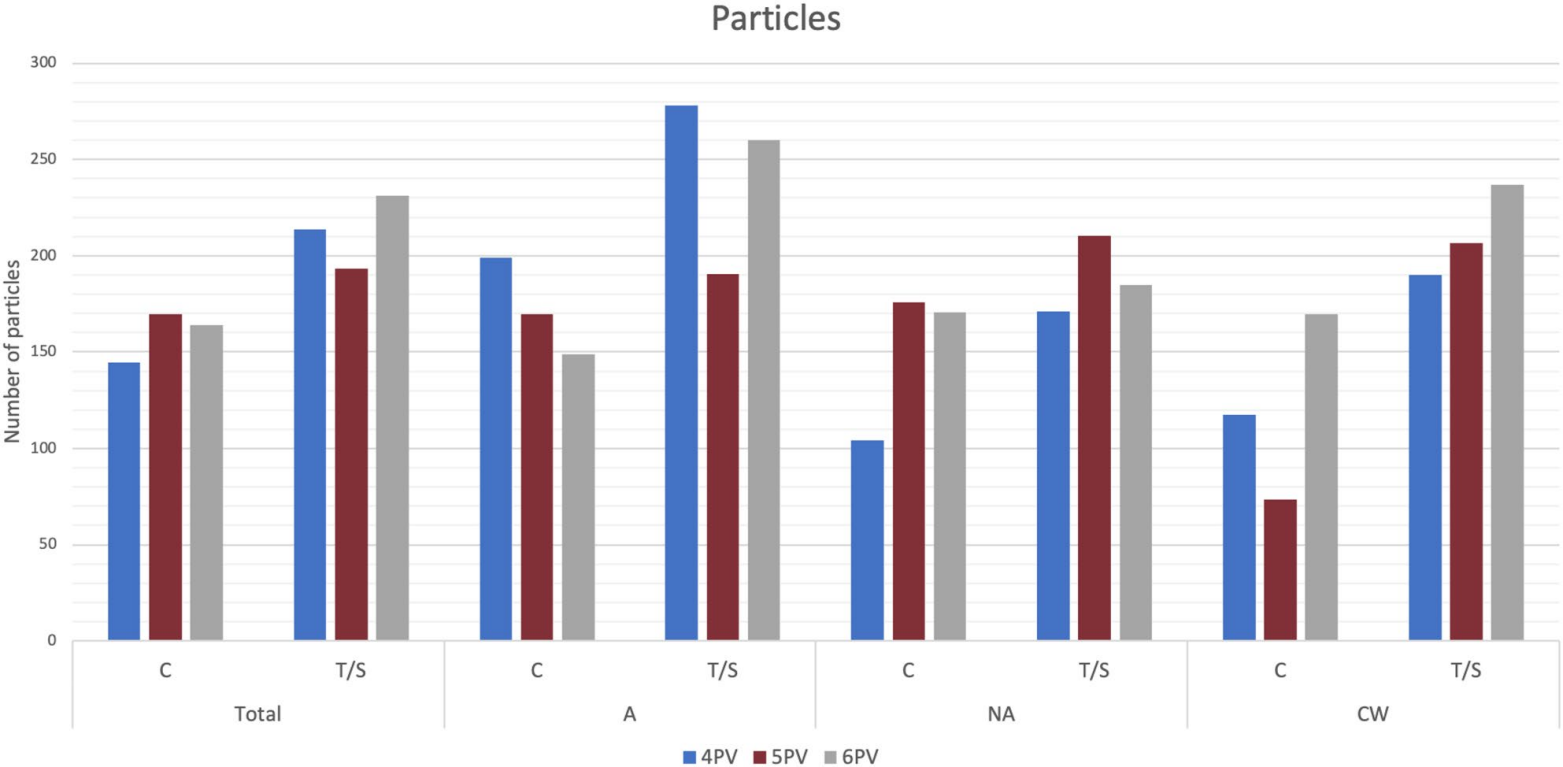
Number of simulated particles for different pulmonary vein configurations for all cases. Number of simulated particles reaching the left atrial appendage for cases with varying number of pulmonary veins (PV), including the control (C) and thrombus/stroke (T/S) groups and different types of LAA and left superior PV alignment. A/NA: aligned/non-aligned; CW: chicken-wing. Blue/red/gray: 4/5/6 PV.

It was also confirmed that the right PV flow contributes more to LAA particles with higher LSPV-LAA misalignment (43.22% and 57.43% in aligned and non-aligned groups, respectively). analysing the influence of each individual PV in the aligned LSPV-LAA group, we could observe that the LIPV was the higher contributor of LAA particles in the control group. On the other hand, in thrombus cases, the left inferior pulmonary vein (LIPV) contribution diminished, while increasing the RIPV input (see Fig. 6). As for the non-aligned group, the opposite behaviour was found, with the RSCPV being the main player in controls, and LIPV in thrombus subjects (see Fig. 5). The chicken-wing LAA group had the same trend as the LSPV-LAA aligned cohort (see Fig. 5).

## Discussion

The work presented in this manuscript focused on studying the influence of the PV configuration on LA blood flow patterns, being the first time these factors were related to potential LAA flow stagnation, and thus thrombus risk. In order to do so, we have created the largest database of patient-specific LA fluid simulations in the literature so far, with more than 130 simulated subjects. Until now, most of the studies had less than 10 processed real LA anatomies, with the exceptions of the preliminary results of our research (Mill et al.,^32^ on 52 cases), and the recents works published by Rigatelli et al., and Fang et al.,^41,46^. Rigatelli et al., conducted a comparative analysis of LA hemodynamics between AF and foramen ovale patients involving a cohort of 60 individuals; meanwhile, Fang et al. performed a FSI analysis of 39 individuals to compare non-stroke and stroke AF patients.

The large studied cohort allowed a detailed analysis of PV morphology and its variability depending on the number and distribution of pulmonary veins. Eleven different PV configurations were found, with cases having from 3 to 7 PV, the most common sub-group being the one with 4 PV, followed by 5 PV subjects (67 and 43 out of 131 cases, respectively). Most of the configurations found in our cohort coincide with those presented in the studies by Marom et al.^47^, Polaczek et al.^48^, and Kato et al.^49^, except the F configuration reported by the latter (with a large deviation of the RSPV). On the other hand, two outlier LA geometries on our cohort, with 3 PV on the left side, with a total of 5 and 7 PV, respectively, were not described in previous research. Criteria used in our work to segment the PV in the CT images (e.g., PV ending cutting plane after the first outgoing branch) could justify differences compared to the literature.

The relation between the PV morphology and AF was also already studied by Polaczek et al.^48^, and Marom et al.^47^. The former did not find any strong relationship between the number the pulmonary veins and AF, but cases with 5 PVs (3 vs. 2 for the right and left sides) (Fig. 1) were more predominant in the AF group. On the other hand, Marom et al.^47^ found this configuration significantly more present in the AF cases. In our study, only analysing AF cases, there were patients with thrombus history in all sub-groups regarding the number of PVs. However, a higher percentage of thrombus vs. control cases were found in LA geometries with 5 and 6 PV. Beyond standard LA morphological parameters, to further investigate the PV configuration and its relation with LA blood flow patterns and thrombus formation risk, we estimated several angles characterising the topological relationship between each PV and its LA environment. In addition, we derived indices from particle-based fluid analysis to estimate the contribution of each PV in flow entering the LAA (Fig. 6).Please check and confirm the inserted citation of Fig. 6 is correct. If not, please suggest an alternative citation. Please note that figures should be cited in sequential order in the text.The citation is ok

As in former investigations^10^, we identified significant differences in controls vs. thrombus cases in ostium characteristics, having larger ostia a higher risk to develop thrombus. However, it is worth noting that the works performed by Fang et al. and Dueñas-Pamplona yielded contrary results^41,42^ although the latter did not have a follow-up on thrombus formation to validate them. Additionally, differences in LA volume and the LAA/LA ratio were found between thrombus and controls. However, as the processed database included CT scans acquired both at end-diastole and end-systole (67 and 63 scans, respectively), which could have a non-negligible impact on LA volumetric indices, the obtained volumetric LA indices could be misleading.

On the other hand, the PV angular parameters and the total number of particles in the LAA were more reliable and significant parameters to differentiate controls from thrombus cases. Of special interest were the $\gamma$ angle, defining the alignment between the LAA and LSPV main directions, and the inclination of the right PV. Despite being the most significant descriptor, the total number of particles, a fluid-based parameter, none other in-silico haemodynamic indices (e.g., ECAP, LAA washout) were helpful to discriminate controls vs. thrombus cases. The most likely reason is the absence of patient-specific boundary conditions in the performed simulations, using the same generic velocity curve at the MV for all subjects (also, very close to the LAA, thus leaving few degrees of freedom near where measurements are taking place). The total number of particles is not heavily affected by the generic boundary conditions (BC) since it mainly depends on the velocity vector field, rather than its magnitude, with the LA geometry having the largest impact. On the contrary, the remaining in-silico haemodynamic indices are highly influenced by the velocity magnitude, which was not found significantly different between controls and thrombus cases in the studied cohort. Particle age did neither yield any significant findings, likely attributed to the fact that particles were continuously introduced into our system until the final time step, and the total number of heart beats studied was limited to one. To obtain more comprehensive insights, it is advisable to leverage more powerful computational resources and conduct additional cardiac beats, while also having a few beats without introducing new particles. This approach would allow for a more thorough assessment of complete washout dynamics.

Although we did not find a direct relation between the number of PV and the probability to suffer a thrombotic event, the PV configuration definitely had a strong impact on blood flow patterns, thus having a certain and complex link with thrombogenesis. For example, the angles (e.g., $\gamma$ angle) and inclinations (e.g., $\phi$ angles of the right side) of the PV fully determined the point of collision of the multiple PV flows in the LA, key to understand how blood patterns evolve and circulate through the LA main cavity and how they reach the LAA, as can be seen in Fig. 1. Our experiments confirmed that the individual PV (and PV side) contributing the most to LAA flow fully depends on the number and configuration of PV. Yaghi et al.^7^ already pointed the relation of LAA angles and blood stasis, but they did not include the PV in their studies. Dueñas-Pamplona et al.,^42^ recently reported the importance of the impact produced by the right PVs on the LA haemodynamics.

The number and PV origin of particles in the LAA were studied in relation to PV sub-groups (i.e., from 3 PV to 7 PV) and the LSPV-LAA main direction alignment. The results showed that, independently on the number of PV, a higher LSPV-LAA alignment was associated to more flow particles entering the LAA. Moreover, a larger LSPV-LAA misalignment made the right PV contribute more to LAA blood flow patterns; with an opposite behaviour, in the LSPV-LAA aligned cases, the LSPV flow collides with the right PV one, bounces off, and goes straight into the LAA ostium.

Analysing all these factors independently, it was not possible to identify trends related to thrombus formation. However, within each sub-group (e.g., aligned LSPV-LAA in 4 PV), the number of particles were associated to the risk of thrombus, among other interesting relations. For instance, in LA geometries with 4 PV, a higher participation of the left side favours the washing of the LAA and, therefore, decreases the probability of thrombus formation. On the other hand, 5 PV LA cases had the contrary behaviour, with a higher right PV flow contribution into LAA particles in controls, as it also was the case for chicken-wing LA morphologies. There were too few samples in 3, 6 and 7 PV groups to draw any relevant conclusions from their analysis.

The chicken-wing LAA morphologies were assessed independently of the LSPV-LAA alignment criteria since the variability in their LAA tip direction, bending angle and ostium position (i.e., anterior/posterior) was huge. In consequence, the number of LAA particles was heterogeneous in the CW cases. Less thrombus cases were found in the CW group, but no specific blood flow pattern could be derived to better understand thrombosis in these morphologies.

In summary, the three most relevant PV characteristics to understand LAA haemodynamics were the number of PV, their angular relationship (especially in the right PV side) and the LSPV-LAA alignment. The combination of these three factors creates a large spectrum of LA blood flow pattern scenarios; arguably certain combinations will be more prone to favour thrombus formation, while others will be more protective.

The large number of possible complex PV configurations can explain the difficulties to find a simple PV topological descriptor linked to thrombogenesis, being then the total number of particles the best individual predictor of thrombus risk. It would be crucial in the future to develop a model combining the analysed factors for a better prediction, also including more advanced ones such as indices quantifying the characteristics of blood flow collision points in the LA^10^. Certainly, the availability of patient-specific BC would improve LA fluid simulations, and more in-silico haemodynamic indices could become significantly different between controls and thrombus cases. More advanced particle models such as discrete phase models with particles interacting with the LA wall and among themselves would improve the realism of the thrombus formation process, and obtain better in-silico haemodynamic indices. Finally, one noteworthy consideration is also the potential impact of having CT images acquired in different phases of the cardiac cycle (e.g., diastole vs. systole), in particular in anatomical measurements. Therefore, in the design of prospective studies, careful attention must be given to the CT acquisition cardiac phase to ensure consistency.

## Conclusions

Thrombus formation in the LAA, which can lead to transient ischaemic attacks, cerebrovascular accidents, and stroke, is influenced by a combination of various factors, including both morphology and blood flow patterns. However, analysing these factors independently does not provide a comprehensive understanding of the underlying patho-physiological mechanisms or enable accurate assessment of individual thrombogenic risk. In this study, we have demonstrated that a joint analysis of morphological parameters, pulmonary vein morphology, and LA haemodynamics can offer improved stratification of patients with and without a history of transient ischaemic attack or cerebrovascular accident. Our findings indicate that relying solely on LAA morphology is insufficient for stratifying these patients, as thrombus cases can occur across various LAA shapes.

The present manuscript represents the largest in-silico study of LA haemodynamics conducted to date. Notably, the total number of particles in the LAA emerged as the most effective individual parameter for distinguishing between the thrombus and non-thrombus groups. Furthermore, we computed novel morphological descriptors of the LA based on the angles formed by the PVs, which proved to be reliable indicators of thrombus formation, particularly when there was alignment between the LAA and LSPV main directions, along with a greater inclination of the right PV. However, more personalised boundary conditions and a comprehensive analysis would yield better and more robust in-silico hemodynamic indices from fluid simulations.

Additionally, our findings suggest a potential protective role of chicken-wing morphologies, consistent with previous literature reports. However, understanding the underlying reasons is complex due to the inherent variability of chicken-wing LAA shapes. Nevertheless, the orientation of the LAA and the configuration of PVs are likely to contribute to this observed effect.

## Methods

### Clinical database

The clinical data of the study were provided by Hospital Haut-Lévêque (Bordeaux, France), including AF patients that underwent a left atrial occlusion (LAAO) intervention and with available pre-procedural high-quality CT scans. 130 patients were available for this study. 61 CT images were acquired in systole and 69 in diastole. Out of the 130, 53 had either a thrombus spotted in the LAA, had a stroke or CVA history; the remaining cases were allocated to the control group. Cardiac computed tomography (CT) studies were performed on a 64-slice dual source CT system (Siemens Definition, Siemens Medical Systems, Forchheim, Germany). Images were acquired using a bi-phasic injection protocol: 1 mL/kg of Iomeprol 350 mg/mL (Bracco, Milan, Italy) at the rate of 5 mL/s, followed by a 1 mL/kg flush of saline at the same rate. The study was approved by the Institutional Ethics Committee, and all patients provided informed consent and we confirm that all experiments were performed in accordance with relevant guidelines and regulations.

### 3D geometrical mesh generation

The left atrial geometries were segmented from the available CT images by a researcher who was not involved in the modelling process, in order to maintain the study blind to the modeller. The LA anatomy was extracted from the CT images using open-source software tools such as Slicer 4.10.1, which employed semi-automatic region-growing techniques to create binary masks of the LA. Subsequently, surface meshes were created using the classical Marching Cubes algorithm, followed by mesh post-processing to correct any irregularities generated by the segmentation step, involving smoothing, manual removal of self-intersecting faces and non-manifold edges. No tubes were added to the pulmonary veins. The cuts of the PVs were made prior to the first bifurcation of the branch originating from the LA, ensuring that the tube was of sufficient length. Software tools like Meshlab 2016.12 and Meshmixer 3.5 were used to build the surface meshes and apply the required mesh manipulations. The Delaunay algorithm was applied, which is available in open-source tools such as Gmsh 4.0.4, creating a tetrahedral mesh. The edge length was set to 1 mm. Additionally, the Netgen software was used for further optimisation of the resulting volumetric meshes. The final volumetric meshes consisted of between 7 and 9 $\times 10^5$ elements, depending on the volume of the LA.

### Morphological descriptors of the left atria and its appendage

**Figure 7:**
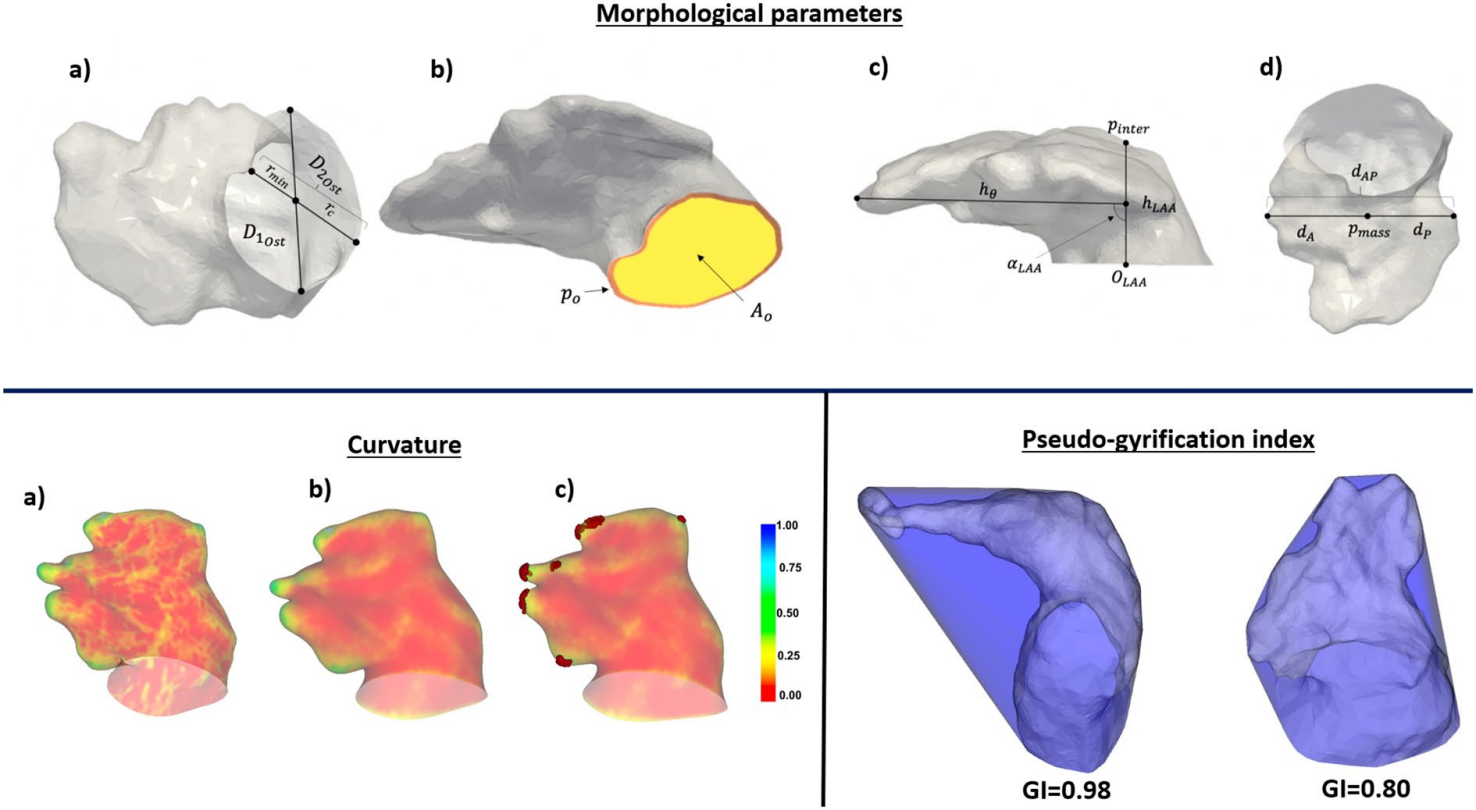
Morphological descriptors of the left atria and its appendage (LAA). Top: (**a**) Maximal (D1_ost_) and minimal (D2_ost_) ostium diameter. (**b**) Ostium perimeter (po) and area (Ao), in orange and yellow, respectively. (**c**) LAA height (h_LAA_), distance to the apex (h_θ_), bending angle ($\alpha _{LAA}$), ostium origin (O_LAA_), and point of mesh intersection (p_inter_). (**d**) LAA anterior (d_A_), posterior (d_p_) and antero-posterior (d_AP_)distances, and centre of mass (p_mass_). Bottom: curvature and gyrification index (GI), left and right, respectively.

The analysed LAA morphological parameters in the study were the following (see Fig. 7):

- the LAA ostium was characterised by its maximum and minimum diameters (D_max_, D_min_, respectively), its area, irregularity and perimeter. The ostium was detected using the method presented in Saiz-Vivó et al., which combines automated and machine learning-based techniques^50^;
- the LA and LAA volumes and its ratio (LAA/LA %);
- the neck height (h_LAA_), the distal point length (h_θ_) and the length of the LAA. The height of the appendage (h_LAA_) was computed by defining the Z axis of the ostium plane going from the origin of the ostium (O$_LAA$) to the surface of the appendage and then measuring the distance between the origin and the point where the axis intersected with the LAA surface (p$_inter$). Similarly, the distance to the apex, was calculated as the maximum length between the mean point of the appendage height axis and every point of the LAA mesh;
- the tortuosity of the LAA, which is the ratio between the neck height and the sum of the distal point length and half of the neck height, represents the irregularity of the LAA in terms of bends and curves; it goes from 0 to 1, with a more irregular the LAA being closer to 1;
- bending of the LAA ($\alpha _{LAA}$ in Fig. 7);
- the mean curvature (^10^) of the LAA. The mesh curvature map was computed with the SLAM python library. The curvature was obtained for each mesh element, where positive values represented concave and convex curvatures, respectively. Subsequently, the mesh was clipped 5 mm inside the LAA from the ostium plane to avoid considering the high curvature obtained at the mesh ostium edge. The mean curvature index was computed as the average of the curvature for each mesh element. Furthermore, from the estimated LAA curvature maps, the LAA lobes were automatically identified through the detection of regions of maximum concave curvature (Fig. 7).
- the 3D gyrification index (GI,^51^). The 3D GI metric was computed by dividing the area of the LAA mesh by the area of its convex hull. In a range from 0 to 1, it provided information on the degree of LAA irregularity, considering LAA meshes more similar to the convex hull (index closer to 1) as more regular.

**Figure 8:**
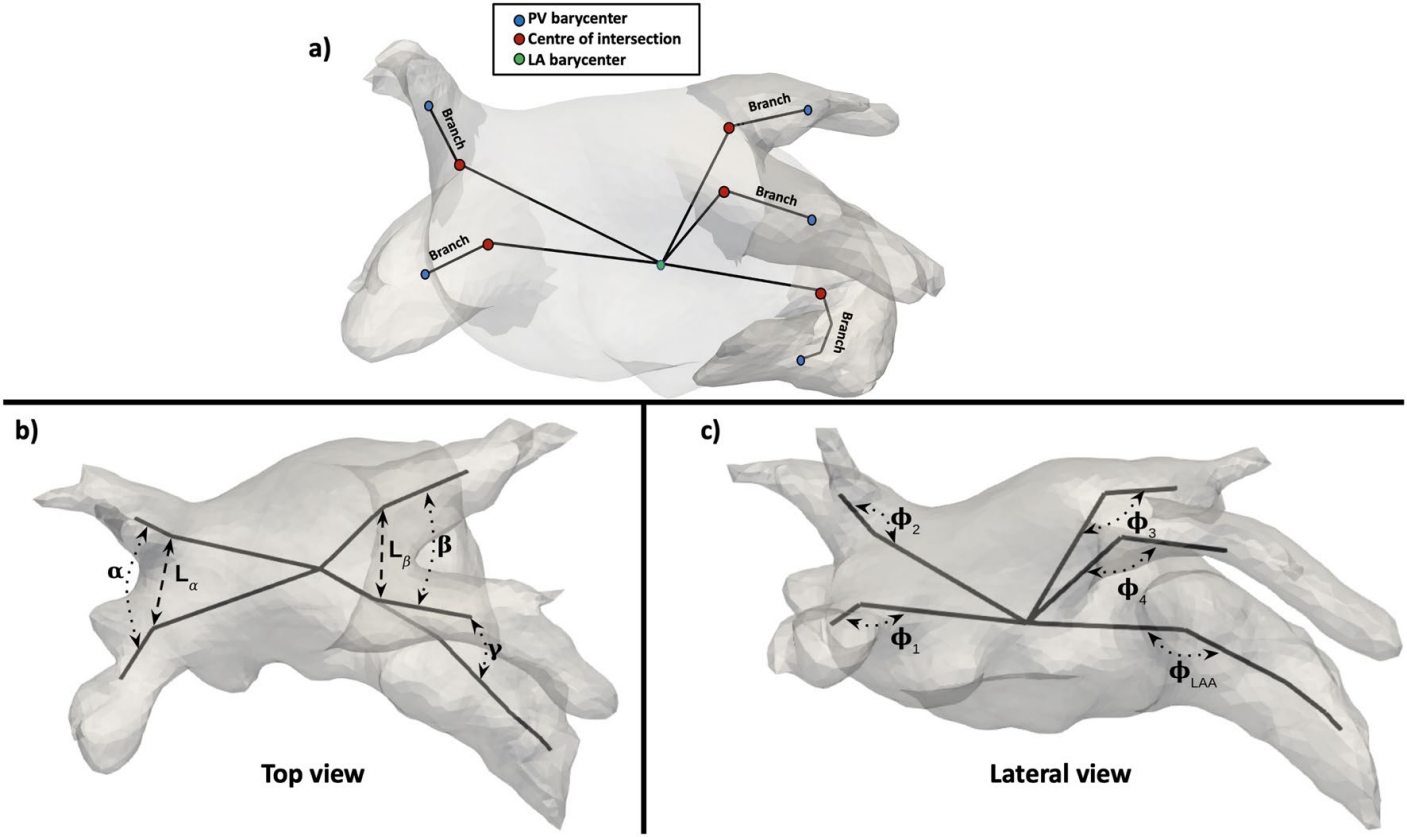
Angles quantifying the configuration of the pulmonary veins in the left atria and the relation with the left atrial appendage (LAA). (**a**) The LA skeleton, depicted in black, is computed linking the barycentres of the different LA sub-structures; (**b**) Top view of the left atrium $\alpha$, $\beta$, $\gamma$ angles, together with the $L_{\alpha }$ and $L_{\beta }$ distances; (**c**) Lateral view of the left atrium with the computed $\phi$ angles.

To study the influence of the PV configuration, the analysed LA geometries were visually classified by the number of pulmonary veins. Subsequently, a set of angles describing the topological relationships between the PV, the LAA, the mitral valve (MV) and the main cavity of the LA, were computed following the approach presented in^32,52^, as illustrated in Fig. 8. The initial step to estimate the LA and LAA angles consisted in the labelling of each pulmonary vein. A diffeomorphic surface registration (using the Deformetrica software^53^) was firstly applied between a previously labelled template LA shape and the rest of the LA meshes. After the surface registration, labels from the LA template were transferred to all cases. Please note that even in cases where there were originally 5 or more pulmonary veins, our analysis was standardised to match the template LA anatomy with 4 main pulmonary veins (see an example in Fig. 2, scenario c).

Therefore, the barycentre of each PV was computed, together with the centre of the ostium (e.g., interface with the LA cavity) of each label, producing a two-point representation of each vein (which it is called a branch). Furthermore, for the LAA angle, an additional landmark was included: the LAA was cut at the barycentre with the normal plane to the LAA centreline, followed by the computation of the barycentre of the outermost half. Finally, the barycentre of the LA main body was added. When linking the extracted landmarks with straight lines, a skeleton-like simplified representation of the LA was obtained, as illustrated in Fig. 8.

From the estimated skeleton and landmarks we computed the following morphological angles and lengths (see Fig. 8):

- $\alpha$ angle: between the right inferior and superior PVs (RIPV and RSPV, respectively);
- $\beta$ angle: between the left inferior and superior PVs (LIPV and LSPV, respectively);
- $L_{\alpha }$ length: between the RIPV and RSPV ostia;
- $L_{\beta }$ length: between LIPV and LSPV ostia;
- $\alpha /\beta$ and $L_{\alpha }/L_{\beta }$, being their respective ratios;
- $\phi$ angles: between the lines linking each PV ostium centre with the PV and LA main body barycentres, characterising the inclination of each PV. From these angles, additional features can be computed such as the mean inclination of all PVs ($1/4(\phi _{1} + \phi _{2}+ \phi _{3} + \phi _{4})$), the right PVs ($1/2(\phi _{1} + \phi _{2})$), and the left PVs ($1/2(\phi _{3} + \phi _{4})$);
- $\phi _{LAA}$ angle: representing the LAA bending angle, being computed between the lines linking the LAA barycentre with its tip and ostium centre;
- $\gamma$ angle: between the LAA ostium and the LSPV, requiring a rigid transformation of the LSPV branch and the LAA to have a common point (e.g., bringing the LAA ostium and the LSPV ostium centres to the same point).

**Figure 9:**
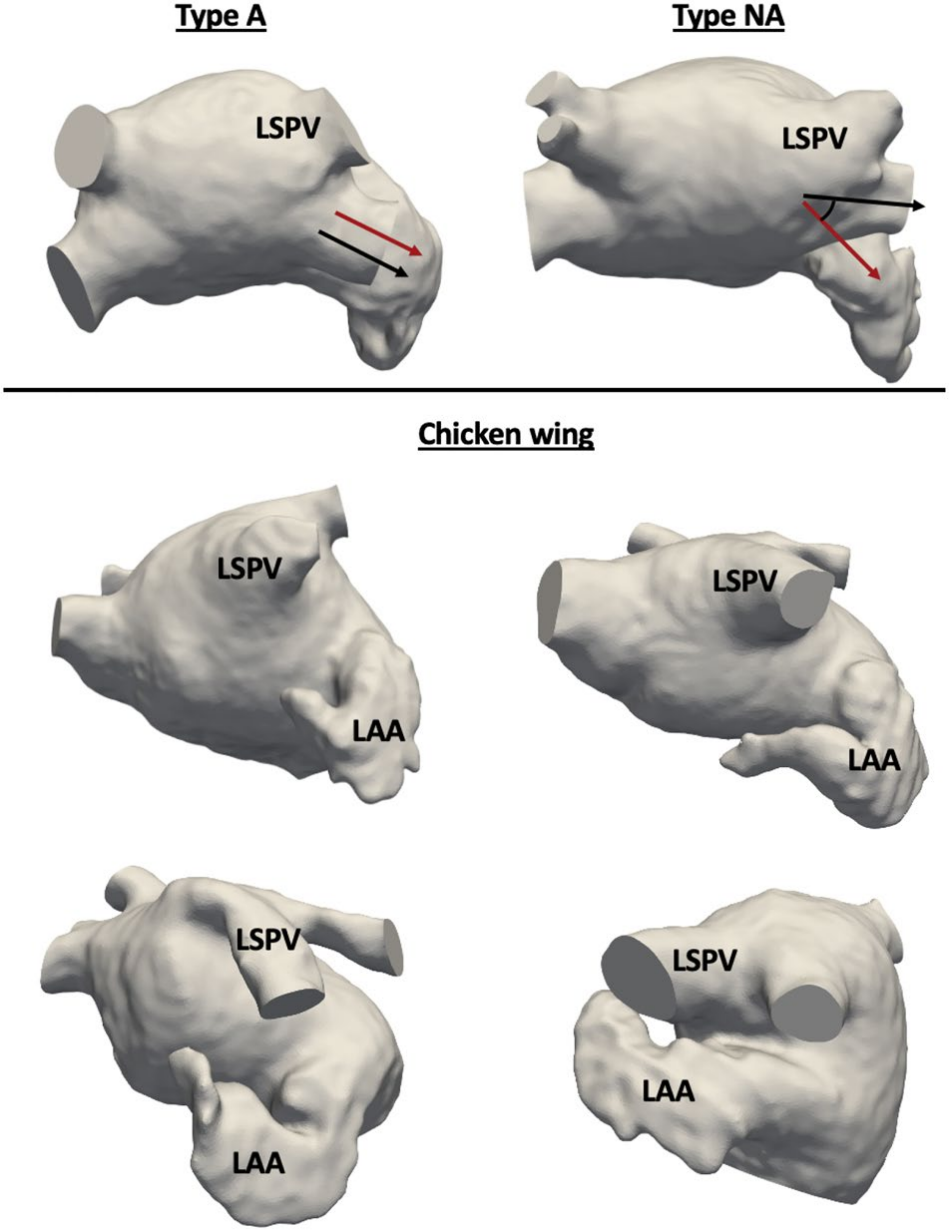
Qualitative classification system based on the alignment between the left superior pulmonary vein (LSPV) and the left atrial appendage (LAA). Top row: the LAA and LSPV main directions are aligned or not (A or NA, respectively). The black and red arrows indicate the main directions of the LSPV and the LAA (i.e., the landing zone between the LAA ostium and neck), respectively. Second and third rows: four different chicken-wing LAA morphologies are shown, demonstrating their variability in localization with respect to the left PVs, bending angles, and tip direction, thus requiring an independent group for the classification.

A novel qualitative classification system was also introduced, based on the alignment between the left superior pulmonary vein and the LAA main directions. As a result, the following classification scheme was established, as illustrated in Fig. 9:

- Group A: alignment of the LAA and LSPV main axes;
- Group NA: non-aligned LAA and LSPV main axes;
- CW group: LAA geometries with a “Chicken-wing” configuration.

Having CW-shaped LAA morphologies into distinct groups was necessary due to the considerable variability observed in ostium positions, bending angles, and tip orientations, which could potentially elucidate the conflicting findings associated with CW shapes. Examples of LA geometries in each category are shown in Fig. 9.

### In-silico computational model

Computational fluid dynamics simulations were conducted, incorporating passive movement of the LA from the longitudinal movement of the mitral valve annulus ring (derived from measurements by Veronesi et al.^54^) within a dynamic mesh approach based on the spring-based method. This approach assumes a comparable longitudinal behaviour in individuals with non-valvular AF and those without AF, as presented in the study by Emilsson et al.^55^.

The ANSYS Fluent Solver 19.2 (ANSYS Inc, United States) was utilised for the simulations, employing the finite volume method. Each simulation spanned three cardiac beats, with the results of the third beat being specifically analysed, while the first two beats were used as a warm-up phase. Blood was characterised as an incompressible Newtonian fluid, with a density of 1060 kg/m^3^ and a dynamic viscosity of 0.0035 Pa s. The time step was set to 0.01 s. To achieve absolute convergence criteria, the residuals for the continuity equations were established at 0.001.

In this study, we applied the same BC setup for all analysed cases, comprising a pressure inlet at the PV and a blood flow velocity outlet at the mitral valve. The pressure waveform was obtained through catheterisation from a patient with AF, while the velocity profile was extracted from a Doppler ultrasound acquisition available in our database. The duration of each cardiac cycle was adjusted based on the information derived from the electrocardiogram (ECG) data. The decision to utilise the velocity profile at the MV was driven by two primary factors. Firstly, ultrasound data was more accessible and with higher quality at the MV that in the pulmonary veins. Secondly, the fluid dynamics implementation of the MV opening/closure is more manageable when imposing a velocity at the MV orifice.

It is important to note that the applied BCs were not specific to individual patients. In addition to the constraint of limited access to patient-specific data, another reason for employing identical pressure and velocity profiles as boundary conditions was to avoid introducing further sources of variation into the model. By maintaining consistency in the boundary conditions, our aim was to primarily investigate the impact of morphological parameters on the haemodynamic patterns of the left atrium, with a particular emphasis on analysing a comparable range of flow characteristics. The profiles applied as BC can be found in Supplementary Figs. A3 and A4.

### Haemodynamic and thrombogenic descriptors

Haemodynamic indices derived from computational fluid simulations, such as blood flow velocities, were estimated by averaging values obtained from the third simulated beat, which encompassed both systolic and diastolic phases. To measure the flow rate, a 2D plane placed beneath the first lobe before the bending of the LAA was selected at the entrance of the LAA. Further details regarding the plane cut can be found in Mill et al.^32^. Flow entering the LAA was considered positive, while the ongoing flow was considered negative. The resulting flow rate curve was then integrated over time to calculate the total volume of blood crossing the selected 2D plane. A zero value in the integration indicated that all flow entering the LAA exited it by the end of the analysed cardiac cycle. Conversely, a large positive value suggested significant blood accumulation within the LAA, potentially indicating flow stagnation. To account for the considerable variation in LAA volume among individuals, the obtained values were expressed as a percentage relative to the LAA volume for each case, representing the proportion of flow volume remaining within the LAA. Additionally, the endothelial cell activation potential (ECAP) was computed based on the obtained fluid simulations, following the methodology described by Di Achille et al.^56^.

In order to evaluate LA haemodynamics and determine the site of collision between the flows from the right and left PV, streamline visualisations were generated. This was achieved by placing 50 seed points in each PV and visually analysing the streamlines during specific time frames throughout the cardiac cycle: (1) at the onset of ventricular systole; (2) just prior to MV opening; (3) when the maximum blood flow velocity was attained during the E wave (early diastole, following MV opening); (4) at the midpoint between the E and A waves; and (5) when the maximum velocity of the A wave was reached (late diastole, just prior to MV closure and the initiation of the next cardiac cycle). This analysis was repeated for two consecutive heartbeats in each case.

Furthermore, to determine the PV origin of blood flow entering the LAA, 100 mass-less particles were distributed among the various pulmonary veins (50 seeds on each side). Those 50 particles were evenly distributed. For example, in cases involving four PVs 25 particles are allocated in each PV. It should be noted that the number of particles allocated to each LA side (50 in total) remained independent of the number of PVs, enabling the assessment of the PV side exhibiting a more predominant blood flow. The particles were tracked throughout the cardiac cycle, computing their pathlines based on the velocity vector field derived from the seed points. After conducting the fluid simulation, the number of particles within the LAA, their PV origin, and their age (i.e., time inside the LA) at the end of the beat were determined.

## Supplementary Information

Supplementary Information.

## Data Availability

The data that support the findings of this study are available from CHU Bordeaux and Universitat Pompeu Fabra but restrictions apply to the availability of these data, which were used under license for the current study, since sensible patient information is involved and so are not publicly available. Data are however available from the authors upon reasonable request and with permission of CHU Bordeaux and Universitat Pompeu Fabra (hubert.cochet@chu-bordeaux.fr and oscar.camara@upf.edu).
