# Supplementary material for: The role of the pulmonary veins on left atrial flow patterns and thrombus formation
Source: Sci Rep. 2024 Mar 11;14:5860. doi: 10.1038/s41598-024-56658-2 (PMC11639444; doi:10.1038/s41598-024-56658-2)
Supplement: Supplementary file 1 — Supplementary Information. [file 41598_2024_56658_MOESM1_ESM.pdf]

Supplementary Information

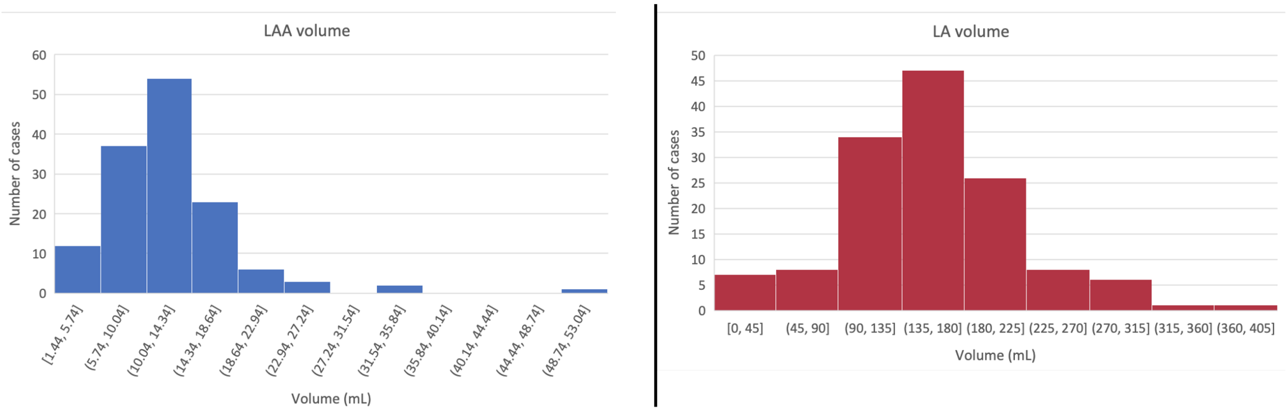

Additional Figure A1. Histograms of the left atrial (LA) and left atrial appendage (LAA) volume distribution (in mL)

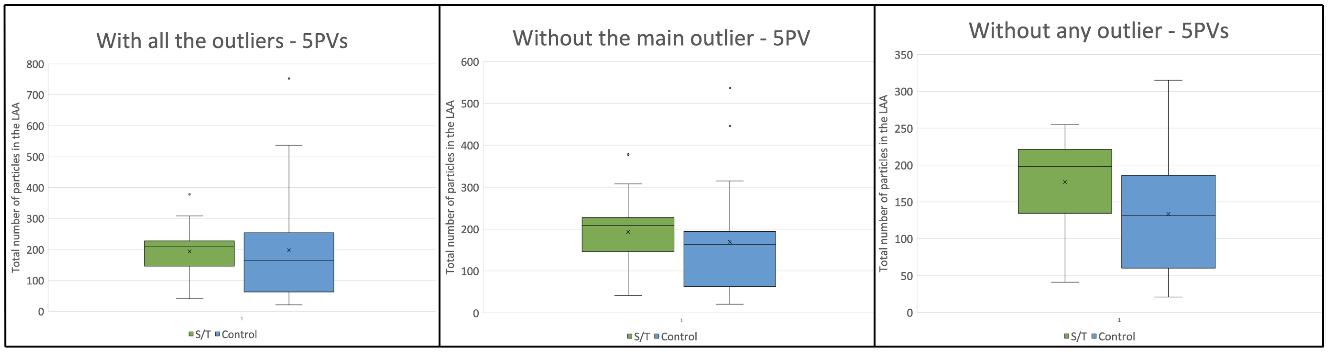

Additional Figure A2. On the left, the box plot for the 5PVs group. At the center, the boxplot with the main outlier (T/S group) removed from the cohort. On the right, the boxplot with all the outliers removed. One case within the control group exhibits a count exceeding 700, significantly deviating from the main distribution of the control group. Consequently, the mean number of particles in the LAA is higher in the control group than in the thrombus group. Upon removing this outlier, the mean undergoes a change (193.5 in the thrombus group vs. 169.5 in the control group). However, it is important to exercise caution in avoiding the fallacy of incomplete evidence. The results presented in the main text are derived directly from the simulations without removing any cases.

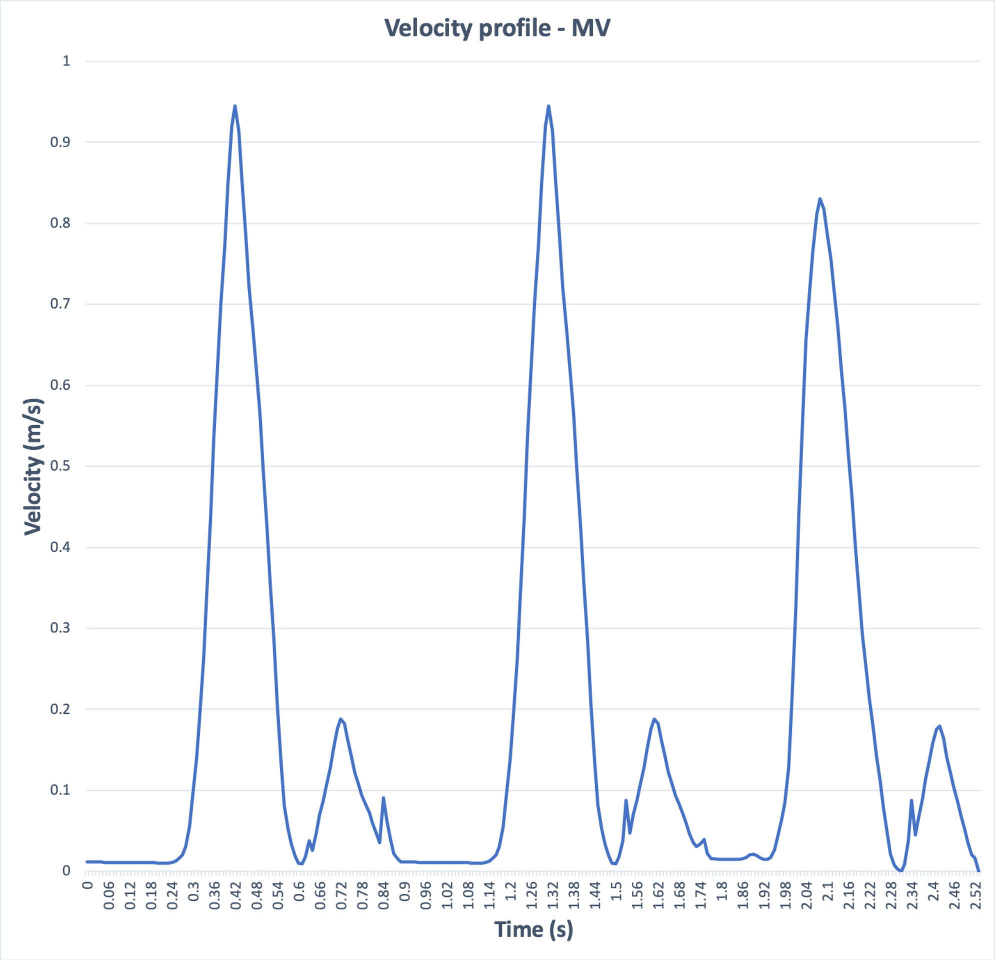

Additional Figure A3. Velocity profile applied at the mitral valve as a Dirichlet condition. The velocity curve was extracted from a US pulsate Doppler from a paroxysmal AF patient. As can be observed the second wave (also know as A wave) is reduced considerably due to the fibrillation.

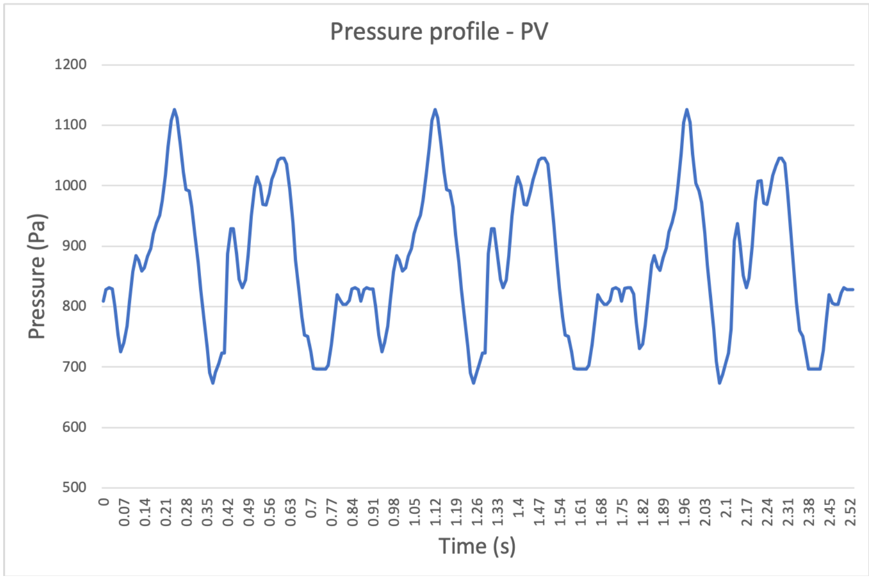

Additional Figure A4. Velocity profile applied at pulmonary veins as a Neumann condition.The curve was measured from AF patient during a procedure using a catheter.

Results for 3 pulmonary veins		
PV origin	C (Type NA)	T/S (Type A)
LCPV (%)	73.96	83.29
RSPV (%)	18.31	15.58
RIPV (%)	7.72	1.13
Left side (%)	73.96	83.29
Right side (%)	26.04	16.71
# Particles	133.33	353.00

Additional Table A1. The cohort of patients with 3 PV comprised merely three control cases and one case with thrombus formation. Notably, the latter case exhibited a high left superior pulmonary vein (LSPV)-LAA alignment, as evidenced by a small γ angle (type A). Furthermore, the thrombus case presented a higher number of flow particles remaining within the LAA at the end of fluid simulations, in contrast to the control cases. Moreover, an overwhelming majority of flow particles in the LAA were coming from the left pulmonary vein (approximately 80%) in these cases, with comparable flow patterns observed between the control and thrombus groups. However, it is worth noting that the control cases had a higher proportion of flow into the LAA coming from the right inferior pulmonary vein (RIPV) due to their lesser alignment of the ostium of the LAA with the LSPV. Pulmonary vein origin of simulated particles reaching the left atrial appendage in cases with 4 pulmonary vein and Type A alignment. C: control. T/S: thrombus or stroke group. L/R: left/right; S/C/I: superior/central/inferior. #: total number. Larger differences between the two groups are shown in bold.

Results for 4 pulmonary veins								
	**Total**		**A**		**NA**		**CW**	
PV origin	C	T/S	C	T/S	C	T/S	C	T/S
LSPV (%)	35.68	31.51	**43.81**	**35.91**	31.56	22.77	32.18	31.02
LIPV (%)	24.27	21.50	22.90	20.75	26.11	17.35	**20.61**	**28.57**
RSPV (%)	24.67	28.45	**18.55**	**26.47**	26.45	34.51	**28.32**	**20.77**
RIPV (%)	16.40	18.71	14.74	17.10	15.86	25.37	19.28	19.64
Left side (%)	59.95	53.01	66.71	56.66	**57.67**	**40.10**	52.79	59.62
Right side (%)	41.07	47.15	33.29	43.57	**42.31**	**59.88**	47.60	40.38
# Particles	144.74	213.50	198.75	278.06	104.15	170.83	117.65	190.00

Additional Table A2. Pulmonary vein origin of simulated particles reaching the left atrial appendage in cases with 4 pulmonary vein and Type alignment. C: control. T/S: thrombus or stroke group. L/R: left/right; S/C/I: superior/central/inferior. #: total number. Larger differences between the two groups are shown in bold.

Results for 5 pulmonary veins								
	**Total**		**A**		**NA**		**CW**	
PV origin	C	T/S	C	T/S	C	T/S	C	T/S
LSPV (%)	30.48	33.40	31.20	33.17	31.67	38.65	31.20	26.28
LIPV (%)	21.60	27.82	**19.30**	**27.58**	25.75	27.71	28.90	28.89
RSPV (%)	17.49	15.99	**20.85**	**12.71**	16.48	15.54	14.20	17.14
RCPV (%)	17.82	10.57	14.06	12.87	**12.51**	**6.56**	11.52	17.06
RIPV (%)	13.52	13.05	14.59	13.67	13.60	11.53	14.18	11.35
Left side (%)	**52.08**	**61.22**	50.50	60.75	59.42	66.36	60.10	55.17
Right side (%)	**47.92**	**38.78**	49.50	39.25	40.58	33.64	39.90	44.83
# Particles	169.45	193.5	207.57(169.45)*	190.55	175.75	210.50	**73.57**	**206.75**

Additional Table A3. Pulmonary vein origin of simulated particles reaching the left atrial appendage in cases with 5 pulmonary vein and Type alignment. C: control. T/S: thrombus or stroke group. L/R: left/right; S/C/I: superior/central/inferior. #: total number. Larger differences between the two groups are shown in bold.

Results for 6 pulmonary veins								
	**Total**		**A**		**NA**		**CW**	
PV origin	C	T/S	C	T/S	C	T/S	C	T/S
LSPV (%)	29.58	27.76	29.65	27.96	31.20	23.76	26.22	29.11
LIPV (%)	25.15	25.17	**37.68**	**23.41**	**13.31**	**34.73**	**23.78**	**17.30**
RSPV (%)	10.72	9.74	10.31	10.12	8.26	11.74	16.46	4.64
RSCSPV (%)	13.99	11.71	9.01	15.57	22.00	6.52	8.93	10.55
RICSPV (%)	10.19	9.02	8.23	6.96	12.08	14.10	9.23	6.96
RIPV (%)	10.35	16.59	5.12	15.97	13.15	9.15	15.24	31.40
Left side (%)	54.73	53.47	67.33	51.37	44.51	58.49	50.00	46.41
Right side (%)	45.27	47.06	32.67	48.63	55.49	41.51	50.00	53.59
# Particles	**164.00**	**231.17**	**149.00**	**260.00**	**170.33**	**185.00**	**169.50**	**237.00**

Additional Table A4. Pulmonary vein origin of simulated particles reaching the left atrial appendage in cases with 6 pulmonary vein and Type alignment. C: control. T/S: thrombus or stroke group. L/R: left/right; S/C/I: superior/central/inferior. #: total number. Larger differences between the two groups are shown in bold.

Results for 7 pulmonary veins		
PV origin	C	T/S
LSPV (%)	44.03	25.00
LIPV (%)	18.91	32.58
RSPV (%)	11.10	16.7
RCPV (%)	7.92	9.09
RIPV (%)	11.82	4.55
RSCSPV (%)	6.18	6.82
RICSPV (%)	7.92	5.30
Left side (%)	62.96	57.58
Right side (%)	37.04	42.42
# Particles	156.00	132.00

Additional Table A5. The analysis of 7 pulmonary vein (PV) cases was limited to four cases, including 3 CW LAA morphologies. The non-CW case, which did not have thrombus, had a high LSPV-LAA alignment and a large left side contribution into LAA particles, following the trend described in cases with a lower number of PV. Finally, it was not possible to find consistent trends in the studied parameters for the CW cases. Pulmonary vein origin of simulated particles reaching the left atrial appendage in cases with 4 pulmonary vein and Type alignment. C: control. T/S: thrombus or stroke group. L/R: left/right; S/C/I: superior/central/inferior. #: total number. Larger differences between the two groups are shown in bold.
